# Effects of Trapezius Muscle Self-Stretching on Muscle Stiffness and Choroidal Circulatory Dynamics: An Evaluation Using Ultrasound Strain Elastography and Laser Speckle Flowgraphy

**DOI:** 10.3390/tomography11070073

**Published:** 2025-06-25

**Authors:** Miki Yoshimura, Takanori Taniguchi, Takeshi Yoshitomi, Yuki Hashimoto

**Affiliations:** 1 Department of Orthoptics and Visual Sciences, Faculty of Health and Medical Sciences, Graduate School of Health and Welfare Sciences, International University of Health and Welfare, Momochihama 2-4-16, Sawara-ku, Fukuoka 814-0001, Japan; 24s1157@g.iuhw.ac.jp (M.Y.); yoshitomi@ihwg.jp (T.Y.); 2 Department of Physical Therapy, Faculty of Medicine, Fukuoka International University of Health and Welfare, Momochihama 3-6-40, Sawara-ku, Fukuoka 814-0001, Japan; taniguchi.tg@ihwg.jp; 3 Department of Orthoptics, Faculty of Medicine, Fukuoka International University of Health and Welfare, Momochihama 3-6-40, Sawara-ku, Fukuoka 814-0001, Japan

**Keywords:** choroidal circulation, stiff shoulder, trapezius muscle, laser speckle flowgraphy, ultrasound strain elastography

## Abstract

Background/Objectives: The relationship between upper trapezius muscle stiffness and choroidal circulatory dynamics remains unclear. This study aimed to examine changes in upper trapezius muscle stiffness and choroidal circulatory dynamics before and after trapezius muscle self-stretching. Methods: Eighteen healthy adults in their 20s (median age ± standard error: 21.0 ± 4.9 years) and eight healthy adults in their 40s (age: 43.0 ± 15.2 years) were included. Intraocular pressure (IOP); systolic, diastolic, and mean blood pressure (BP); heart rate (HR); ocular perfusion pressure (OPP); and salivary alpha-amylase (sAA) activity—as an indicator of autonomic nervous system function—were measured at baseline and after trapezius muscle self-stretching. Upper trapezius muscle stiffness was assessed using ultrasound strain elastography, whereas choroidal circulation was evaluated using laser speckle flowgraphy to determine the mean blur rate (MBR), a relative measure of macular blood flow velocity. Results: Significant reductions in systolic and mean BP; OPP; sAA activity; and MBR were observed after trapezius muscle self-stretching in both groups; however, no significant changes were found in IOP and HR. A significant decrease in upper trapezius muscle stiffness was observed after self-stretching only in the 20-year-old group. Conclusions: In healthy adults in their 20s and 40s, trapezius muscle self-stretching may enhance parasympathetic nervous system activity, resulting in decreased systemic and choroidal circulatory parameters. However, the reduction in muscle stiffness observed only in younger participants suggests that short-term self-stretching may be less effective in reducing trapezius muscle stiffness with advancing age.

## 1. Introduction

Stiff shoulder is a common musculoskeletal disorder, with a reported prevalence of 30–50% [1]. In recent years, attention has been drawn to stiff shoulders associated with prolonged computer use [2,3]. Stiffness of the upper trapezius muscle has been identified as a primary cause of neck and shoulder pain. Increased stiffness of the upper trapezius muscle has been reported in individuals with neck and shoulder disorders compared with healthy individuals [4,5]. Furthermore, patients with chronic stiff shoulders have been shown to exhibit four times greater upper trapezius muscle activity during computer work compared with healthy individuals [6]. These findings highlight the importance of evaluating and managing upper trapezius muscle stiffness in individuals with neck and shoulder complaints. Recently, the elastography function of ultrasound imaging devices has been used to assess upper trapezius muscle stiffness [7,8]. Elastography provides an index of muscle stiffness by calculating the strain ratio or measuring shear wave velocity [9,10]. Several interventions for alleviating upper trapezius muscle stiffness, including heat therapy [11], ultrasound therapy [12], and self-stretching [13,14], have been reported.

Conversely, a stiff shoulder is associated with eye strain, which manifests as dry eyes, blurry vision, and ocular discomfort or pain [15]. Tasks requiring high visual acuity, such as computer work, increase muscle activity in the trapezius muscles, resulting in heightened discomfort and fatigue in the neck and shoulder regions [15]. Therefore, in the management of stiff shoulders, eye fatigue should be considered along with upper trapezius muscle stiffness. Eye strain has been attributed to abnormalities in the anterior segment and extraocular muscles, such as reduced tear production [16,17] and diminished accommodative ability [18,19]. However, no studies to date have examined the effects of stiff shoulders on the choroid, which is located in the posterior segment of the eye and accounts for approximately 90% of total ocular blood flow. The choroid is highly sensitive to autonomic nerve system activity due to its limited capacity for autoregulation [20,21,22]. Laser speckle flowgraphy (LSFG) is a noninvasive method used for assessing ocular blood flow [23,24]. This technique provides the mean blur rate (MBR), a relative index of blood flow velocity. Choroidal MBR increases under sympathetic nervous system dominance and decreases when the parasympathetic activity is elevated [25,26,27,28,29,30].

Self-stretching activates the parasympathetic nervous system and reduces stress hormone levels [31,32]. In addition, therapeutic back massage in women in their 20s, evaluated using shear wave elastography, resulted in reduced trapezius muscle stiffness [33]. However, no previous studies have examined the changes in upper trapezius muscle stiffness using strain elastography in conjunction with autonomic nervous activity and choroidal circulation following self-stretching of the trapezius muscle. Therefore, this study aimed to investigate changes in autonomic nervous function, upper trapezius muscle stiffness, and both systemic and choroidal circulatory dynamics before and after trapezius muscle self-stretching. The hypothesis proposed that self-stretching would reduce muscle stiffness, enhance parasympathetic nervous system activity through the relaxation response, and subsequently lower both systemic circulation and choroidal blood flow.

## 2. Materials and Methods

### 2.1. Participants

This study was approved by the Ethics Committee of Fukuoka International University of Health and Welfare (approval ID: 20-fiuhw-022) and was conducted in accordance with the tenets of the Declaration of Helsinki. Written informed consent was obtained from all participants. This prospective intervention study (pre–post comparison test) included the right eyes of 18 healthy adults in their 20s and 8 healthy adults in their 40s (Table 1). Individuals with systemic conditions such as ophthalmic, orthopedic, and neurological diseases; who were unable to undergo accurate examinations; and who were unable to provide informed consent for participation in the study were excluded. Participants were recruited through voluntary response, a non-probability sampling method. Each participant underwent best-corrected visual acuity, intraocular pressure (IOP), blood pressure (BP), heart rate (HR), and autonomic nervous system assessments; fundus photography; strain elastography; and LSFG.

### 2.2. Self-Stretching of the Trapezius Muscle

**Figure 1:**
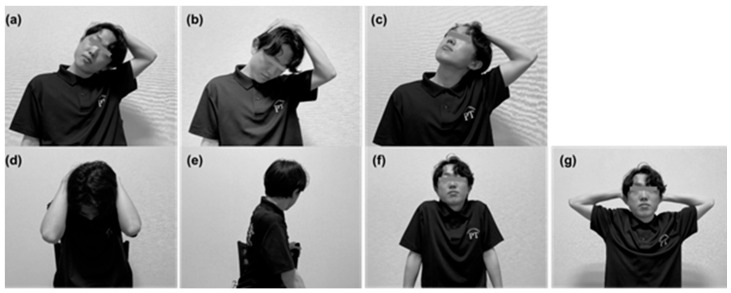
Self-stretching exercises for the trapezius muscle. (**a**) Neck lateral flexion. (**b**) Neck flexion with lateral flexion and ipsilateral rotation. (**c**) Neck extension with lateral flexion and contralateral rotation. (**d**) Neck flexion. (**e**) Trunk twisting with ipsilateral neck rotation. (**f**) Scapular elevation and depression. (**g**) Scapular abduction and adduction.

Self-stretching of the trapezius muscle was performed based on previously described protocols and included seven exercises (Figure 1): Figure 1a: neck lateral flexion, Figure 1b: neck flexion with lateral flexion and ipsilateral rotation, Figure 1c: neck extension with lateral flexion and contralateral rotation, Figure 1d: neck flexion, Figure 1e: trunk twisting with ipsilateral neck rotation, Figure 1f: scapular elevation and depression, and Figure 1g: scapular abduction and adduction [13]. Exercises Figure 1a–e involved static and passive stretching, with each stretch held for 20 s and repeated for five sets. Exercises Figure 1f,g were active scapular movements, performed for 20 repetitions per set, across three sets. The entire stretching program lasted approximately 15 min.

### 2.3. Intraocular Pressure and Systemic Hemodynamics

IOP, systolic BP (SBP), diastolic BP (DBP), and HR were measured before and immediately after trapezius muscle self-stretching. IOP was assessed using a non-contact tonometer. The mean BP (MBP) was calculated from the SBP and DBP, whereas the ocular perfusion pressure (OPP) was derived from the MBP and IOP.

### 2.4. Autonomic Nervous System Assessment

Salivary alpha-amylase (sAA) activity, which reflects plasma norepinephrine concentrations, was used as a noninvasive marker of sympathetic nervous system reactivity. A saliva collection paper was placed under the tongue. Saliva was collected for approximately 30 s, and sAA activity was measured.

### 2.5. Ultrasound Strain Elastography

The stiffness of the upper trapezius muscle was measured using the ultrasound strain elastography function of an ultrasound image analyzer (LOGIQ P9; GE Healthcare, Tokyo, Japan). Strain elastography evaluates tissue deformation in response to applied pressure and calculates relative stiffness based on this deformation. Participants were placed in the prone position and instructed to remain relaxed to minimize the effects of muscle contraction.

**Figure 2:**
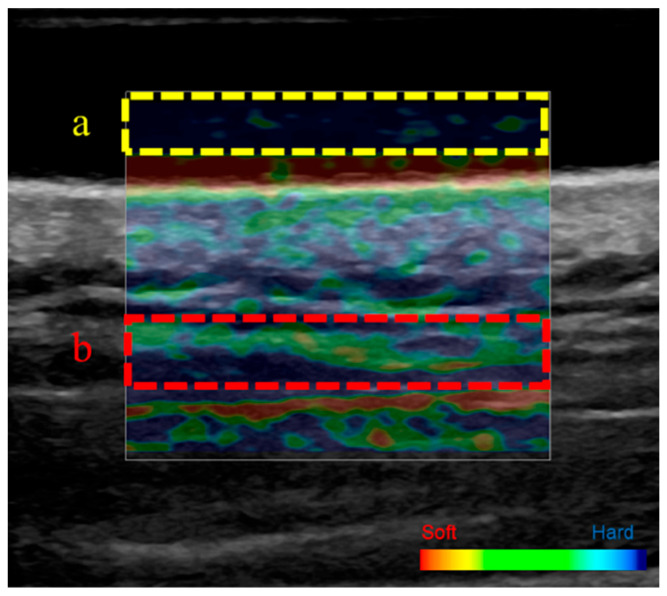
Ultrasound strain elastography image and regions of interest (ROIs). ROI of the acoustic coupler gel (**a**) and ROI of the upper trapezius muscle (**b**). Red indicates softer areas, whereas blue indicates harder areas.

The measurement site was determined using a tape measure in accordance with previous studies [34], specifically at the midpoint between the 7th cervical spinous process and the acromion. The skin surface was marked to ensure consistent probe placement and prevent measurement errors. An acoustic coupling gel pad (Echo Gel PAD EP-S-10, 100 × 100 × 10 mm; Yasojima, Kobe, Japan) was applied to the marked site. Measurements were performed using a linear probe. To ensure consistent compression speed and force, the compression waveform displayed on the monitor was continuously observed, and data were recorded once the waveform stabilized. The upper trapezius muscle and acoustic coupling gel were identified on the color-displayed strain elastography image, and each was designated as a region of interest for strain measurement (Figure 2). In accordance with previous studies [35], muscle stiffness was defined as the strain ratio between the upper trapezius muscle and acoustic coupling gel. The strain ratio was calculated using the elastography function of the ultrasound device, based on a scale ranging from 0 (soft) to 6 (stiffest): strain ratio = scale of upper trapezius muscle fibers/scale of acoustic coupling gel. A high strain ratio indicated greater muscle stiffness, whereas a low strain ratio indicated reduced stiffness. All measurements were performed three times consecutively by the same examiner, and the mean value was used for analysis.

### 2.6. Laser Speckle Flowgraphy

LSFG-NAVI (Softcare Ltd., Fukuoka, Japan) was used to measure the hemodynamics of the posterior fundus. This system uses an 830 nm diode laser to illuminate the fundus and detect the movement of red blood cells in the deep choroidal vessels [25,26,27,28,29,30]. The laser speckle technique enables quantitative and reproducible measurements of blood flow velocity [23,24], with each acquisition completed in approximately 4 s. Measurements were performed three times at baseline and after self-stretching. To assess changes in choroidal blood flow velocity, large retinal vessels in the macular area were excluded from the analysis. For each healthy participant, the same measurement region was automatically selected by the LSFG Analyzer software (version 3.0.47; Softcare Ltd., Fukuoka, Japan), ensuring consistency with the baseline measurement site. Changes in average MBR, a quantitative index of relative blood flow velocity, were evaluated as the percentage relative to the baseline value (set as 100%).

### 2.7. Statistical Analyses

The Wilcoxon signed-rank test was used to assess the changes in IOP, SBP, DBP, HR, MBP, OPP, sAA activity, trapezius muscle stiffness, and MBR before and after self-stretching of the trapezius muscle. Comparisons between the 20- and 40-year-old groups were conducted using the Mann–Whitney U test or Fisher’s exact test, as appropriate. A *p* value of <0.05 was considered significant. All statistical analyses were performed using BellCurve for Excel (v 4.08; Social Survey Research Information Co., Ltd., Tokyo, Japan). Results are expressed as the median ± standard error. Additionally, Cohen’s d was calculated as a post hoc analysis to estimate the statistical power of the study based on the sample size. The sample size calculation was carried out using G*Power (v 3.1.9.6), based on a Wilcoxon Mann–Whitney test comparing two groups, with an assumed effect size of 0.80 and a significance level of 5%.

## 3. Results

Eighteen eyes of healthy volunteers in the 20-year-old group (thirteen women and five men) and eight eyes of healthy volunteers in the 40-year-old group (one woman and seven men) were examined (*p* = 0.009). The median ages were 21.0 ± 4.9 years (20–24 years) and 43.0 ± 15.2 years (41–47 years), respectively (*p* < 0.001). The median refractive errors were −2.63 ± 0.64 D (+0.50 to −5.75 D) and −2.50 ± 0.77 D (+4.00 to −8.00 D), respectively (*p* = 0.696) (Table 1). Post hoc analysis showed that the sample size provided a statistical power of 42.1% (1 − β = 0.421).

### 3.1. IOP, Systemic Hemodynamics, and sAA Activity

The changes in IOP, SBP, DBP, MBP, HR, and sAA activity are presented in Table 2 and Table 3. Compared with baseline, both the 20- and 40-year-old groups exhibited significant decreases in SBP (*p* = 0.002 vs. *p* = 0.030), MBP (*p* < 0.001 vs. *p* = 0.017), OPP (*p* = 0.004 vs. *p* = 0.035), and sAA activity (*p* = 0.038 vs. *p* = 0.027) (Table 2 and Table 3) after trapezius muscle self-stretching. In the 20-year-old group, the DBP significantly decreased after trapezius muscle self-stretching (*p* = 0.014). However, no significant changes were observed in IOP (*p* = 0.170 vs. *p* = 0.262) and HR (*p* = 0.239 vs. *p* = 0.068) in either group compared with baseline.

### 3.2. Stiffness of the Upper Trapezius Muscle

**Figure 3:**
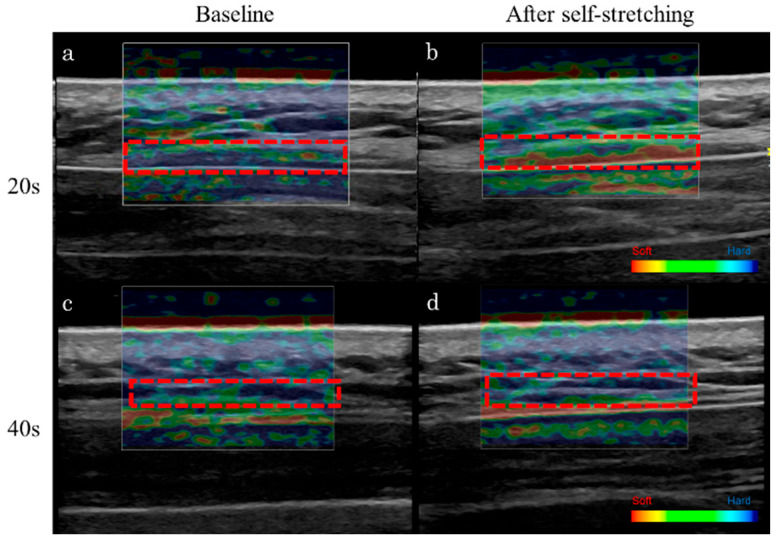
Changes in the strain ratio of the upper trapezius muscle after self-stretching assessed using ultrasound strain elastography. The 20-year-old participant exhibited a decrease in muscle stiffness following self-stretching (**b**) compared with baseline (**a**). However, the 40-year-old participant showed minimal change in muscle stiffness following self-stretching (**d**) compared with baseline (**c**). Red indicates softer tissue, whereas blue indicates harder tissue.

The changes in the stiffness of the upper trapezius muscle are presented in Table 2 and Table 3. In the 20-year-old group, trapezius muscle stiffness significantly decreased after self-stretching (baseline, 0.5 ± 0.1 vs. after stretching, 0.4 ± 0.1; *p* < 0.001) (Table 2 and Table 3, Figure 3). In the 40-year-old group, no significant change was observed in muscle stiffness after stretching (baseline, 0.4 ± 0.1 vs. after stretching, 0.5 ± 0.2; *p* = 0.179) (Table 2 and Table 3, Figure 3).

To evaluate the reproducibility of measurements by a single examiner, the stiffness of the trapezius muscle was measured three times, and the intraclass correlation coefficient (ICC) was calculated. The intra-examiner reliability was high (ICC = 0.80), indicating excellent correlation consistency.

### 3.3. Choroidal Blood Flow Velocity

**Figure 4:**
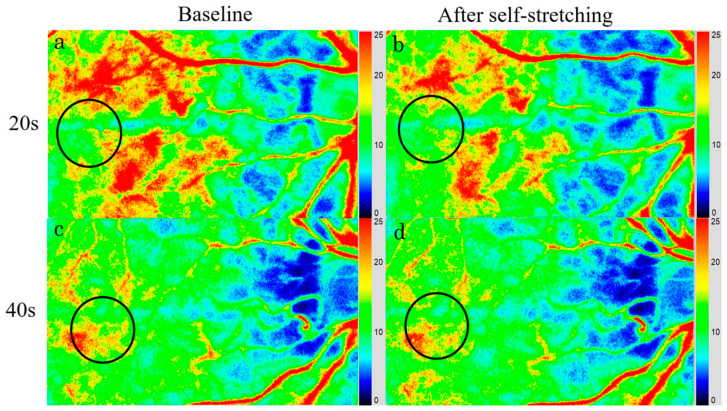
Laser speckle flowgraphy (LSFG) images of 20-year-old and 40-year-old participants at baseline (**a**,**c**) and after upper trapezius muscle self-stretching (**b**,**d**). Following the self-stretching, the MBR decreased by 11.3% in the 20-year-old group (**b**) and by 4.6% in the 40-year-old group (**d**), compared with baseline values (**a**,**c**). On composite color map image of LSFG, MBR within circles was analyzed. Blue indicates lower MBR, whereas red indicates higher MBR.

The MBR values are presented in Table 2 and Table 3. At baseline, the median macular MBRs were 13.9 ± 3.5 in the 20-year-old group and 10.7 ± 3.9 in the 40-year-old group. After upper trapezius muscle self-stretching, the median MBRs decreased to 12.4 ± 3.1 and 9.8 ± 3.6 in the 20- and 40-year-old groups, respectively (Table 2 and Table 3, Figure 4). The macular MBR significantly decreased by −9.3 ± 2.3% in the 20-year-old group and by −8.6 ± 3.1% in the 40-year-old group (*p* < 0.001 vs. *p* = 0.011) after stretching (Table 2 and Table 3, Figure 4).

## 4. Discussion

Choroidal blood flow velocity, along with SBP, DBP, and MBP, as well as sAA activity, significantly decreased after trapezius muscle self-stretching in the 20-year-old and 40-year-old groups. By contrast, upper trapezius muscle stiffness significantly decreased after self-stretching in the 20-year-old group; however, no significant changes were observed in the 40-year-old group. To the best of our knowledge, this study is the first to examine the stiffness of the upper trapezius muscle and the choroidal circulatory dynamics before and after trapezius muscle self-stretching.

In this study, muscle stiffness significantly decreased after self-stretching of the trapezius muscle. This result is consistent with previously reported changes in muscle properties resulting from stretching. Static stretching changes the viscoelastic properties of the muscle–tendon unit and reduces muscle stiffness [36]. Additionally, stretching improves local blood flow and facilitates the removal of metabolic byproducts, which contributes to the reduction in muscle tension [37]. Furthermore, stretching exerts an inhibitory effect on sympathetic nervous system activity [32], which may further contribute to the decrease in muscle tone. The decrease in muscle stiffness observed in this study may be attributed to the aforementioned physiological mechanisms. A significant reduction in trapezius muscle stiffness was noted among participants in their 20s following self-stretching; however, no significant change was observed in participants in their 40s. This difference may be influenced by several physiological and morphological factors. One key factor is the age-related alteration in the properties of muscle and connective tissue. In individuals in their 40s, age-related changes such as muscle fiber degeneration and atrophy, increased intramuscular connective tissue, and enhanced collagen cross-linking have been reported [38]. These factors may alter the viscoelastic properties of muscle and reduce its plasticity in response to stretching stimuli. Additionally, age-related declines in muscle blood flow and vascular reactivity have been documented [39], potentially leading to insufficient metabolic changes and intramuscular pressure reduction following stretching. Furthermore, aging may reduce the sensitivity of sensory receptors such as muscle spindles and Golgi tendon organs, potentially weakening the reflexive muscle relaxation response to stretching [40]. Consequently, the ability to induce muscle hypotonia through stretching may be reduced in older individuals compared with younger individuals.

In addition, significant decreases in choroidal blood flow velocity, SBP, DBP, MBP, and sAA activity were observed after trapezius muscle self-stretching in the 20-year-old and 40-year-old groups. Stretching is known to promote parasympathetic nervous system dominance, which induces a relaxed physiological state, and can improve stress hormone levels [31,32]. In the present study, a significant decrease in sAA activity—an indicator of sympathetic nervous system activity—was observed following self-stretching. Parasympathetic dominance leads to vasodilation, resulting in decreased BP [20,21]. Furthermore, the choroid demonstrates limited autoregulatory capacity and is directly influenced by the autonomic nervous system activity [20,21,22]. Therefore, following foot immersion in warm water or periocular skin warming, parasympathetic nervous system activity increases as a result of relaxation, leading to a decrease in choroidal blood flow velocity due to reduced systemic circulatory dynamics [29,30]. Neck and shoulder pain have been reported to be associated with ocular strain, including symptoms such as dry eyes, blurred vision, and eye discomfort or pain [15].

The present study has some limitations. Firstly, the sample size was small, resulting in a statistical power of 42.1%, which falls below the generally accepted threshold of 80.0%. Additionally, the participants were relatively young, and the majority of individuals in their 40s were men. Previous studies have reported gender differences in trapezius muscle stiffness [41], whereas findings regarding gender differences in autonomic nervous system activity remain inconclusive [42,43,44]. Secondly, this study examined only the effects of short-term self-stretching. Thirdly, factors such as dry eye, accommodation, and subjective symptoms of eye strain were not examined. Future studies should include a larger and more diverse sample, taking into account gender differences and including older participants. The long-term effects of self-stretching should also be investigated. Moreover, further research is warranted to assess changes in trapezius muscle stiffness and choroidal circulatory dynamics in conjunction with chronic or severe shoulder stiffness, accommodative function, and subjective visual symptoms. A more comprehensive evaluation of autonomic nervous system activity—such as the inclusion of heart rate variability measurements—should also be considered.

In conclusion, the results of the present study suggest that self-stretching of the trapezius muscle exerts a relaxing effect, leading to increased parasympathetic nervous system activity and influencing both systemic and choroidal circulatory dynamics. However, with increasing age, short-term self-stretching may not produce measurable changes in trapezius muscle stiffness, which can be assessed using ultrasound strain elastography.

## Figures and Tables

**Table 1:** Characteristics of participants and ocular biometric parameters in the 20s and 40s groups.

	20 s Group	40 s Group	*p* Value
Sex (women/men)	13/5	1/7	0.009
Age	21.0 ± 4.9	43.0 ± 15.2	0.593
RE (D)	−2.63 ± 0.64	−2.50 ± 0.77	0.696

SE, standard error; RE, refractive error; D, diopter. Mann–Whitney U test. Fisher’s exact test. Median ± SE.

**Table 2:** Changes in biometric parameters and general ocular factors before and after upper trapezius muscle self-stretching in participants in their 20s.

	Baseline	After Self-Stretching	*p* Value (Wilcoxon Signed-Rank Test)
IOP (mmHg)	15.0 ± 3.4	14.0 ± 3.3	0.170
SBP (mmHg)	109.5 ± 26.2	106.0 ± 25.3	0.002 **
DBP (mmHg)	72.0 ± 16.8	70.3 ± 16.3	0.014 *
MBP (mmHg)	84.7 ± 19.9	82.6 ± 19.3	<0.001 ***
HR (bpm)	76.0 ± 18.0	74.0 ± 17.6	0.239
OPP (mmHg)	46.0 ± 11.0	45.3 ± 10.7	0.004 **
sAA (KU/L)	3.0 ± 1.5	3.0 ± 1.0	0.038 *
MBR	13.9 ± 3.5	12.4 ± 3.1	<0.001 ***
MBR (%)	100.0	90.7 ± 21.3	<0.001 ***
Strain ratio	0.5 ± 0.1	0.4 ± 0.1	<0.001 ***

SE, standard error; IOP, intraocular pressure; SBP, systolic blood pressure; DBP, diastolic blood pressure; MBP, mean blood pressure; HR, heart rate; OPP, ocular perfusion pressure; sAA, salivary amylase activity; MBR, mean blur rate. median ± SE. *** *p* < 0.001, ** *p* < 0.01, * *p* < 0.05.

**Table 3:** Changes in biometric parameters and general ocular factors before and after upper trapezius muscle self-stretching in participants in their 40s.

	Baseline	After Self-Stretching	*p* Value (Wilcoxon Signed-Rank Test)
IOP (mmHg)	15.5 ± 5.3	14.4 ± 5.2	0.262
SBP (mmHg)	130.0 ± 45.8	122.0 ± 44.0	0.030 *
DBP (mmHg)	86.5 ± 30.1	85.5 ± 29.6	0.575
MBP (mmHg)	102.8 ± 35.3	99.6 ± 34.4	0.017 *
HR (bpm)	77.8 ± 28.8	77.3 ± 27.6	0.068
OPP (mmHg)	57.6 ± 20.0	56.6 ± 19.5	0.035 *
sAA (KU/L)	9.5 ± 4.2	4.0 ± 1.7	0.027 *
MBR	10.7 ± 3.9	9.8 ± 3.6	0.011 *
MBR (%)	100.0	91.4 ± 32.3	0.011 *
Strain ratio	0.4 ± 0.1	0.5 ± 0.2	0.179

SE, standard error; IOP, intraocular pressure; SBP, systolic blood pressure; DBP, diastolic blood pressure; MBP, mean blood pressure; HR, heart rate; OPP, ocular perfusion pressure; sAA, salivary amylase activity; MBR, mean blur rate. median ± SE. * *p* < 0.05.

## Data Availability

The dataset supporting the findings of this study is available from the corresponding author upon reasonable request.

## Abbreviations

The following abbreviations are used in this manuscript:

IOP	intraocular pressure
BP	blood pressure
SBP	systolic blood pressure
DBP	diastolic blood pressure
MBP	mean blood pressure
HR	heart rate
OPP	ocular perfusion pressure
sAA	salivary alpha-amylase
MBR	mean blur rate
RE	refractive error
D	diopter
ICC	intraclass correlation coefficient
LSFG	laser speckle flowgraphy
